# Extracellular Vesicles and Immune System Function: Exploring Novel Approaches to Colorectal Cancer Immunotherapy

**DOI:** 10.3390/biomedicines12071473

**Published:** 2024-07-03

**Authors:** Antonio Biondi, Marco Vacante, Roberta Catania, Giuseppe Sangiorgio

**Affiliations:** 1 Department of General Surgery and Medical-Surgical Specialties, University of Catania, Via Santa Sofia 78, 95123 Catania, Italy; abiondi@unict.it (A.B.); roby.catania@hotmail.it (R.C.); 2 Unit of Internal Medicine Critical Area—ARNAS Garibaldi, Piazza Santa Maria di Gesù, 5, 95124 Catania, Italy; marcovacante@yahoo.it; 3 Department of Biomedical and Biotechnological Sciences, University of Catania, Via Santa Sofia 97, 95123 Catania, Italy

**Keywords:** extracellular vesicles, immunotherapy, colorectal cancer

## Abstract

This review explores the emerging role of extracellular vesicles (EVs) in modulating immune system function and their application in novel cancer immunotherapy strategies, with a focus on colorectal cancer (CRC). EVs, as carriers of bioactive molecules, have shown potential in enhancing immune responses and overcoming the limitations of traditional therapies. We discuss the biogenesis, types, and functional roles of immune cell-derived EVs, their interactions with cancer cells, and their implications in antitumor immunity. Challenges such as tumor heterogeneity and immune evasion are addressed, alongside the promising therapeutic prospects of EV-based strategies. This comprehensive analysis underscores the transformative potential of EVs in cancer treatment paradigms.

## 1. Introduction

Cancer remains a leading cause of death globally, with traditional treatments like chemotherapy and radiotherapy causing severe side effects. In the last decade, cancer immunotherapies, which activate the immune system to fight cancer, have emerged as a promising alternative [1,2]. The immune system plays a vital role in maintaining physiological balance and can act against cancer [3,4]. Immunotherapy, targeting only cancer cells, offers milder side effects by modulating immune functions. It includes successful approaches like immune checkpoint antibodies, monoclonal antibodies, vaccinations, and chimeric antigen receptor (CAR)–T cell therapies, showing significant promise in clinical trials [5]. The immune system, with its innate and adaptive components, defends against tumors and pathogens, with some cells providing long-term memory to prevent recurrence [6,7,8]. Despite challenges like tumor heterogeneity, immunotherapies, such as dendritic cell modulation and CAR–T cell therapies, are being explored, though they face limitations [9,10,11]. Recently, the focus has shifted to extracellular vesicles (EVs), which offer new avenues for cancer treatment due to their ability to carry therapeutic molecules and cross biological barriers [12,13]. EVs, especially those derived from immune cells, are being investigated for their potential in cancer therapy, highlighting their role in intercellular communication and tumor progression [14,15]. This review explores the structural attributes and significant impacts of EVs derived from innate and adaptive immune cells, providing novel perspectives for advancing cancer diagnosis and treatment in the future.

## 2. Characteristics of Extracellular Vesicles

**Figure 1:**
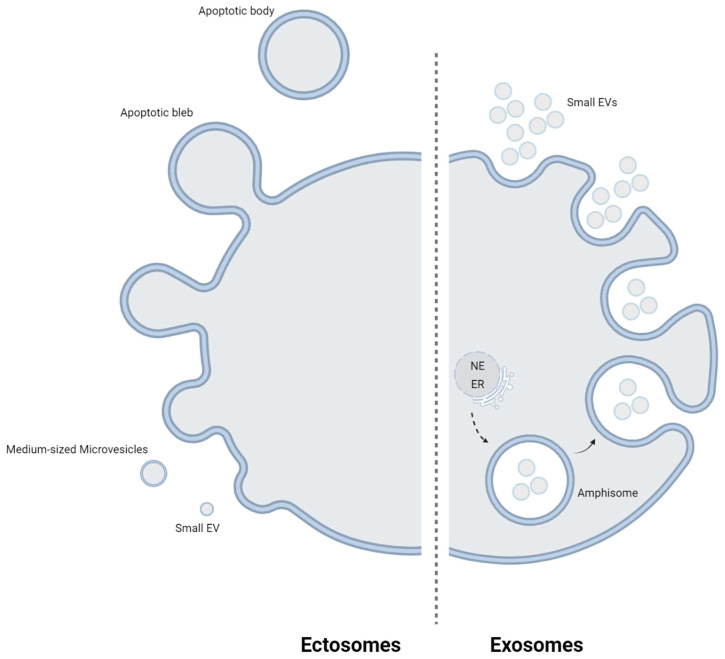
Main types of EVs. The two main types of EVs, exosomes and ectosomes, are distinguished by their biogenesis. Exosomes, originating from endosomes, are small EVs released via exocytosis from multivesicular bodies and amphisomes. Recent data suggest other endomembrane systems like the ER and NE may also be involved. In contrast, ectosomes form through plasma membrane budding and blebbing, comprising small-sized EVs, medium-sized microvesicles, and larger apoptotic bodies. EVs, extracellular vesicles; ER, endoplasmic reticulum; NE, nuclear envelope. This figure was created by the authors with BioRender.com, accessed on 16 May 2024.

EVs exhibit diverse phospholipid membrane-enclosed structures, manifesting differences at unexpected biophysical, biochemical, and functional levels [16,17,18]. These EVs are divided into two main types, exosomes and ectosomes, based on their distinct biogenesis (Figure 1). Exosomes are small EVs released through the exocytosis of multivesicular bodies and amphisomes, with recent data suggesting the involvement of additional endomembrane systems, such as the endoplasmic reticulum [19] and nuclear envelope [20], in their biogenesis [17,21,22]. In contrast, ectosomes are generated by plasma membrane budding and blebbing, with some carrying endosomal cargo components, including small-sized EVs, medium-sized microvesicles, and larger apoptotic bodies [18]. Nevertheless, there are currently no definitive molecular markers available for their various biogenetic routes, prompting the proposal of operational terms to differentiate between EV types based on their biophysical or biochemical properties [18,21]. These EVs are identified in diverse biological fluids, including blood, urine, saliva, and cerebrospinal fluid, and display size heterogeneity due to the diverse releasing cell types and distinct biogenetic pathways [23,24]. The most prevalent among them are small EVs, ranging from 50 to 150 nm in diameter, followed by medium-sized EVs (100–1000 nm) and larger EVs (≥1 μm) [14,25,26,27,28,29]. Table 1 provides an overview outlining the key characteristics of each vesicle. EVs, generated through various cell death mechanisms, intersect with viral egress [30], secretory autophagy, the cellular senescence-associated secretory phenotype, and the DNA damage response [31]. They play pivotal roles in homeostatic processes, including the rapid removal of molecules, cell maturation, adaptation to environmental changes, and the activation of blood clotting. Moreover, EVs modulate other cell functions by delivering intercellular signals through surface proteins, encapsulated cargo molecules, lipids, and glycans [24]. Both cytokines and EVs serve as mediators of intercellular communication, with cytokines associating with EVs as internal or external cargo.

## 3. Isolation and Characterization

International guidelines for isolating and characterizing EVs are regularly updated to enhance the purity and quantity necessary for research and clinical applications [18]. Challenges include differentiating EVs from other nanoparticles like lipoproteins and newly discovered exomeres and supermeres [40,41], and the lack of specific inhibitors for EV biogenesis. Common isolation methods include ultracentrifugation, immunoaffinity capture, and size-dependent techniques [15,18,42,43]. EVs are typically identified using methods like Western blotting, flow cytometry, and various microscopy techniques such as scanning electron microscopy, transmission electron microscopy, and cryoelectron microscopy, dynamic light scattering, atomic-force microscopy, resisting pulse sensing, and nanoparticle tracer analysis [44,45,46]. Characteristics of each of these isolation methods are summarized in Table 2. The isolation and characterization of EVs can significantly impact the diagnosis and therapeutic development of colorectal cancer (CRC) in several ways. EVs contain specific molecular signatures from their cells of origin, making them valuable for the non-invasive early detection of CRC when isolated from blood or other biofluids [47,48]. High-purity EV isolation methods, such as size exclusion chromatography or immunoaffinity capture, reduce contamination from non-EV proteins, leading to more specific and sensitive biomarker detection [49,50]. Additionally, characterizing the cargo of isolated EVs, including proteins and miRNAs, can help classify CRC subtypes and inform prognosis [51].

## 4. EVs in Innate Immunity

EVs play crucial roles in both innate and adaptive immune responses, with various immune cells like monocytes, neutrophils, and dendritic cells (DCs) releasing EVs that contribute to inflammatory processes [52,53]. Natural killer (NK) cells, for instance, use EVs to induce apoptosis in tumor cells, while macrophages and DCs utilize EVs for antigen presentation and stimulating immune responses [54]. Neutrophils produce EVs that exhibit bactericidal activities and can influence macrophage polarization. Studying EVs and innate immunity has significant implications for diagnosing and developing therapies for CRC. Understanding innate immune mechanisms in CRC could lead to preventive strategies, as chronic inflammation increases CRC risk [55,56]. Profiling the innate immune composition of tumors may help stratify patients for specific treatments and predict outcomes [55]. Additionally, innate immune cells and pathways offer potential targets for new CRC therapies, aiming to activate antitumor immune responses [55,57].

NK cells are crucial for organ immunosurveillance, defending against cancer and pathogens through germline-encoded surface receptors that regulate their cytotoxic activity [58]. These receptors include both activating and inhibitory types, which respond to changes in the extracellular environment [59]. Normally, inhibitory receptors like KIRs and NKG2A/CD94 bind to human leukocyte antigen molecules, controlling NK cell activity to prevent damage to healthy cells [60]. During early tumorigenesis or infection, activating receptors such as NKp46, NKp30, NKp44, NKG2D, and DNAM-1 facilitate the formation of immunological synapses, enabling NK cells to destroy abnormal cells. In advanced stages, dominated by tumor cells or viruses, inhibitory receptors become essential to restrain NK cell activity [61,62,63]. NK-derived EVs inherit these surface receptors and can induce apoptosis in tumor cells. However, tumor cells may reduce the expression of active receptors, limiting NK cells’ effectiveness [64,65]. These EVs also carry cytotoxic proteins for direct cancer cell destruction and molecules that support cellular homing, adhesion, and immune activation, enhancing their impact on peripheral blood mononuclear cells and increasing CD56+ NK cells [63,66]. Additionally, NK-derived EVs containing tumor suppressor miRNA-186 show cytotoxic effects against neuroblastoma cell lines [67]. A novel microfluidic system that uses patient-specific NK cells and NK-derived EVs has shown potential for targeting circulating tumor cells, offering new avenues for personalized NK-based immunotherapies with diagnostic and prognostic capabilities [68].

Macrophages exhibit diverse functional phenotypes in response to microenvironmental signals and infiltrate tumors as tumor-associated macrophages, influencing tumor progression [69,70]. The spectrum between pro-inflammatory M1 and anti-inflammatory M2 macrophages determines their impact, with M1 promoting tumor cell phagocytosis and M2 supporting tumor growth and metastasis [71]. Macrophages interact with EVs, absorbing antigens and delivering them to T cells through receptor–ligand interactions [72]. Macrophage-derived EVs exhibit diverse functions influenced by parental cell phenotypes, stimulatory factors, lysosomal functions, autophagy, aging, and the hypoxic tumor microenvironment [73,74,75]. These EVs mediate cell-to-cell communication, facilitating the exchange of miRNAs, long non-coding RNAs (lncRNAs), and proteins [76]. For instance, miRNA-223 induces macrophage differentiation, while miRNA-16-5p from M1 macrophages enhances T cell-dependent immune responses [77]. LncRNAs like PVT1 and AFAP1-AS1 modulate the tumor microenvironment and contribute to pathogenesis. Macrophage-derived EVs also carry protein effectors like ERAP1 and CCL3, enhancing phagocytic functions [78]. Notably, vesicle-mimetic nanovesicles from M1 macrophages can repolarize M2 macrophages to M1, augmenting the antitumor efficacy and suppressing tumor growth through pro-inflammatory cytokine release [79].

DCs play a crucial role in bridging innate and adaptive immune responses by capturing and presenting tumor-associated antigens to initiate antitumor immune responses [80,81]. DCs release EVs, which are small lipid vesicles utilized to stimulate antitumor immune responses in both preclinical and clinical settings [82]. These EVs contain essential components such as CD1 proteins for lipid antigen cross-presentation, tumor antigen peptide–MHC complexes, costimulatory factors, and ligands for NK cell receptors [83]. Additionally, DC-derived EVs carry heat-shock proteins, metabolic enzymes, and various RNAs, including miRNAs, which facilitate intercellular communication and induce post-translational modifications [84]. These EVs mediate cell-to-cell interactions and miRNA exchange, with their contents being influenced by the maturation stage of the DCs [85]. Studies have shown that EVs can directly present tumor antigen–MHC complexes to T cells, resulting in a potent antitumor effect [86]. Furthermore, DC-derived EVs deliver tumor antigens to other DCs, promoting antitumor immunity [87]. The activation of both T and B cells, especially CD8+ T cells, is observed, and strategies that enhance DC maturation significantly increase IFN-γ-producing CD8+ T cells and IL-2 levels [82]. CD4+ T cell propagation is extensively initiated by the vesicle TAA-MHCII complex, particularly when DCs are loaded with a protein rather than a peptide antigen [82,88,89]. 

Neutrophil-derived EVs exist as distinct subtypes: neutrophil-derived trails (NDTRs) generated by migrating neutrophils, and neutrophil-derived microvesicles (NDMVs) produced at inflammation sites [90,91,92]. The production mechanisms are influenced by the immune environment, emphasizing adhesion molecule interactions. These EVs share characteristics such as surface markers, stimulating factors, and bactericidal activity, utilizing ROS- and granule-dependent mechanisms for bacterial elimination [93]. NDTRs require integrin-mediated interactions, while NDMV production relies on the PI3K pathway. Notably, NDMVs prominently express CD16, while NDTRs express PSGL-1 and Fcγ type III receptor at higher levels; both are efficiently taken up by monocytes [94]. NDTRs induce pro-inflammatory M0 macrophage polarization, whereas NDMVs prompt an anti-inflammatory phenotype [93,95]. Differential miRNA expression analysis reveals that NDTRs contain pro-inflammatory miRNAs (miR-4454, miR-1260, miR-7975, and miR-1285), while NDMVs harbor anti-inflammatory miRNAs (miR-451a, miR-150, and miRNA-126), showcasing neutrophils’ adaptive miRNA packaging based on the immune context [93]. Moreover, neutrophil-derived EVs, including granules, exhibit antimicrobial properties, providing defense against pathogens. With a short lifespan and ease of handling, these EVs hold promise for drug delivery applications.

## 5. EVs in Adaptive Immunity

Adaptive immune cell-derived EVs play crucial roles in T and B cell development, antigen presentation, and immune synapse formation [96]. As lymphocytes and antigen-presenting cells are key players in immune defense against pathogens and cancer, understanding how their EVs contribute to immunosuppression and antitumor responses is of significant interest. These EVs can modulate immune responses, making them potential targets for cancer immunotherapies. Research indicates that CRC patients experience significant changes in the numbers and function of their adaptive immune cells, such as reduced T cell counts and increased immunosenescence in advanced stages [97]. Furthermore, combining EV-based therapies with immunotherapies could enhance treatment efficacy by targeting tumor cells and modulating the immune response simultaneously [98,99].

The development of lymphocytes and the role of CD4+ T cell-derived EVs are pivotal in cancer dynamics. Studies indicate that adaptive immune cells, especially T cells, are critical in the development and progression of CRC. The interplay between immune surveillance and tumor-promoting inflammation mediated by various T cell subsets can significantly impact disease outcomes [97,100]. Lymphocytes, including effector T cells (CD4+ helper and CD8+ cytotoxic) and B cells, secrete EVs that influence tumor progression and the tumor microenvironment [54]. CD4+ T cell-derived EVs, in particular, may be pivotal in cancer immunotherapy as they can suppress cytokine production and effector T cell responses. These EVs carry molecules like CD73, CD25, and CTLA-4; notably, CD73+ EVs convert extracellular adenosine-5-monophosphate into adenosine, which suppresses activated T cell responses and inhibits cytokine production [24]. Additionally, these EVs contain miRNAs with proapoptotic or antiproliferative effects, further modulating effector T cell activity. These characteristics highlight their potential in targeted cancer treatments, leveraging their regulatory capacities to enhance therapeutic outcomes [101].

T cell EVs, primarily derived from thymic epithelial cells, play a role in lymphocyte and thymocyte development due to their cargo. Maturation protein cargo induces the maturation of single-positive thymocytes, while carrying antigens to thymic dendritic cells enables EVs to participate in the negative selection of lymphocytes with self-antigen specificity, contributing to antigen presentation [102,103]. These EVs also transfer miRNAs from T cells to antigen-presenting cells unidirectionally [104]. EVs released by B cells directly present antigens to T cells, as they can contain functional peptide–MHC complexes [105], and when attached to dendritic cells, this process becomes more efficient, increasing T cell activation [106]. Antigen presentation can also occur indirectly when peptide–MHC-positive EVs are internalized and processed by antigen-presenting cells [107]. EVs are involved in cross-presentation, contributing to immunity against viruses and tumors due to the presence of MHC class I complexes for CD8+ T cells [96]. Studies have shown that various CD8+ T cell subtype-derived vesicles contribute to tumor immunosuppression [54], but they can also promote tumor progression. For instance, in melanoma, in vitro-activated CD8+ T cell-derived EVs activate ERK and NF-kB, increasing MMP9 expression and cancer cell invasion [108]. Furthermore, there is evidence indicating that EVs derived from lymphocytes promote tumor progression in esophageal cancer by triggering the epithelial-to-mesenchymal transition (EMT) and facilitating metastasis [109]. Studies on CRC have also underscored the significant role of EVs in its progression, metastasis, and immune modulation. For instance, EVs can initiate the epithelial–mesenchymal transition, stimulate angiogenesis, and create pre-metastatic niches, thereby supporting metastasis [49,98].

EVs significantly influence B cell differentiation by mediating the exchange of CD24 among B cells, particularly during their immature primary status [110]. The secretion of EVs from B cells is driven by TCR-MHC class II interactions, enabling these vesicles to facilitate CD4+ T cell [111] and cytotoxic T lymphocyte activation and responses [112]. However, the role of B cell-derived EVs is complex, as high levels can induce CD4+ T cell apoptosis, and their impact on antitumor responses is controversial due to their potential to inhibit CD8+ T cell responses, thereby reducing the efficacy of chemotherapy [111]. This inhibition is partly possible because B cell-derived EVs contain CD39 and CD73, enzymes that convert the ATP released by tumor cells post chemotherapy into adenosine, thus attenuating the treatment’s effects [113]. Furthermore, EVs play a crucial role in the functions of the immune synapse, a critical exchange site formed between lymphocytes (T cells, B cells, or NK cells), antigen-presenting cells [104], and targets [114]. This synapse facilitates the transfer of EV cargo and miRNAs, enhancing intercellular communication. Notably, the synaptic exosomal transfer between T cells and B cells involves various miRNAs that silence genes crucial for immune responses, such as PTEN and BIM, impacting the germinal center reaction and B lymphocyte functions [115,116]. This miRNA exchange also supports antibody production and germinal center development [117]. Additionally, B cells form synapses with follicular dendritic cells, which are potential targets for B cell EVs that transfer MHC class II molecules, essential for B cell differentiation. This intricate network of interactions and effects underscores the multifaceted roles of EVs in immune regulation and responses [118].

## 6. EVs in Inflammation

Inflammation and EVs play critical roles in the pathogenesis, progression, and treatment of CRC. Inflammatory processes, particularly in chronic conditions like inflammatory bowel disease, are pivotal in CRC development. Persistent inflammation, driven by cytokines, chemokines, and growth factors, fosters an environment conducive to tumor formation by promoting cellular proliferation, enhancing survival mechanisms, and triggering genetic mutations in colon and rectal cells [119,120,121]. CRC activates the NF-κB and STAT3 signaling pathways, perpetuating the production of pro-inflammatory cytokines that sustain tumor growth and survival [120,121]. These inflammatory processes also recruit immune cells to the tumor microenvironment, where their activation state can either enhance antitumor responses or inadvertently facilitate tumor progression and metastasis [122,123]. Pro-inflammatory cytokines such as IL-6, TNF-α, and IL-1β further contribute to creating a tumor-promoting microenvironment by influencing cellular behaviors like migration and invasion [120,121]. Additionally, inflammatory signals can induce the epithelial–mesenchymal transition, where epithelial cells acquire characteristics that enhance their ability to migrate and invade surrounding tissues, thereby promoting metastasis [49,123].

EVs play versatile roles in inflammation, acting as carriers of pro-inflammatory bioactive lipid mediators such as eicosanoids that influence chemotaxis [124,125]. Neutrophil-derived EVs transfer arachidonic acid, activating COX1 enzymes, which induce thromboxane A2 production and contribute to neutrophil extravasation [126]. Similarly, platelet-derived EVs containing the enzyme 12-lipoxygenase produce 12-hydroxyeicosatetraenoic acid, affecting neutrophil behavior in inflammatory arthritis [127]. EVs also interact with the extracellular matrix, playing a role in forming chemotactic gradients for migrating inflammatory cells [128]. In some pathological conditions such as sepsis, EVs display both pro-inflammatory and anti-inflammatory effects [129]. Pro-inflammatory actions include the release of cytokines, damage-associated molecular patterns (DAMPs), and mitochondrial DAMPs, which influence macrophage polarization, T helper cell differentiation, and leukocyte chemotaxis. Conversely, some EVs exhibit anti-inflammatory effects through downregulating complement factors, acute phase signaling, reducing leukocyte chemotaxis, and inhibiting adhesion molecule expression on endothelial cells, involving the release of CD14 from macrophage-derived EVs and the suppression of NF-κB activation in LPS-stimulated macrophages [130]. Additionally, EVs from different types of cell death elicit distinct immune responses: necroptotic cells release EVs that induce pro-inflammatory cytokine secretion by macrophages [131], while inflammasome activation during pyroptosis leads to the release of exosomes carrying pro-inflammatory miRNAs and interferon-β [132]. Furthermore, soluble innate-immunity mediators like C-reactive protein (CRP) are transported by EVs, further influencing chemotaxis and the inflammatory response [133].

## 7. Immunomodulation

EVs from both immune and non-immune cells play a pivotal role in immune regulation, influencing the pathology of inflammatory, auto-immune, and infectious diseases. Recently, they have emerged as potential targets for therapies aimed at modulating the immune system; EVs carry various immune regulation molecules, including immune checkpoint molecules like CTLA4, PDL1, FASL (CD95L), and ectoenzymes CD39 and CD73, which induce immunosuppression [134]. For instance, melanoma cells use exosomal PDL1 to promote immunosuppression, highlighting EVs as a tool for identifying responders to anti-PD1 therapy in melanoma treatment [96,135]. EV-mediated immune regulation is crucial for preventing autoimmunity and plays a significant role in gestational immunology. Treg cell-derived vesicles contain miRNAs that increase IL-10 production and decrease IL-6 production in DCs [136], while syncytiotrophoblast-derived EVs contribute to immunosuppression at the fetal–maternal interface [137]. Stem cell-derived EVs also regulate immunity by inhibiting lymphocytes, NK cells, DCs, and monocytes/macrophages through apoptosis induction, cell suppression, and the downregulation of molecular expression and mechanisms. This multifaceted role of EVs in immune regulation underscores their potential in developing therapies for various clinical conditions with an immune component, including tumors [138].

## 8. Antimicrobial Responses

The gut microbiota significantly influences the development, progression, and treatment outcomes of CRC [139,140]. Dysbiosis, an imbalance in gut bacteria commonly found in CRC, involves an overgrowth of harmful bacteria and a reduction in beneficial ones [141]. This imbalance contributes to chronic inflammation in the gut, a well-established risk factor for CRC [142]. Moreover, interactions between pathogens and immunocompromised hosts pose challenges due to impaired immune responses and the potential emergence of antimicrobial-resistant strains [143,144]. Inflammatory molecules produced by the pathogens contribute to cellular changes that promote tumor initiation and progression [139]. Pathogenic bacteria can evade immune detection, thereby supporting tumor growth and metastasis [145]. Conversely, certain bacteria can activate immune responses against tumor cells, influencing the course of disease progression [141,142].

EVs play a crucial role in modulating microbial infections by enabling immune cells to recognize EVs from microbes such as bacteria, fungi, and parasites, thereby inducing the host’s innate immune response [146,147,148]. These vesicles are more effective than soluble peptides in transferring antigens between antigen-presenting cells and can sometimes protect microbes from immune attacks [96,134]. Microbial EVs trigger pro-inflammatory effects through microbial-associated patterns recognized by pattern recognition receptors (PRRs) and carry antigens that stimulate immune and inflammatory responses. For instance, EVs from macrophages infected with pathogens like *Mycobacterium tuberculosis, Salmonella typhimurium*, or *Toxoplasma gondii* carry antigens that activate macrophages via Toll-like receptors [149]. *Staphylococcus aureus* EVs are recognized by PRRs, leading to their degradation [150], while *Plasmodium falciparum* EVs activate the cytosolic stimulator of interferon genes pathway in monocytes [151].

EVs also offer protection from immune attacks, with viral components from the *Picornaviridae* and *Herpesviridae* families being shielded from immune recognition by encapsulation in EVs [152]. Microbial EVs defend against the complement system and protect pathogens from complex-mediated lysis [153]. For example, *Escherichia coli*-derived EVs guard against microbial peptides and antibiotics [154].

Neutrophil-derived EVs contribute to both pro-inflammatory and anti-inflammatory responses, with their production being influenced by the complement receptor Mac-1 [155,156]. These EVs can enhance neutrophil phagocytic capacity and release elongated neutrophil-derived structures (ENDs) during inflammation. ENDs, formed by neutrophils rolling on the endothelium, contain the S100A8-S100A9 complex, promoting leukocyte recruitment and cytokine secretion [96]. Studies comparing healthy individuals and sepsis patients show significantly lower levels of ENDs in the plasma of healthy subjects [36].

## 9. Antitumor Activity

EVs secreted by all cells, including tumor cells, are pivotal in cancer research due to their roles in modulating antitumor responses and their potential as diagnostic and therapeutic tools. The tumor microenvironment (TME), comprising the cellular and noncellular components surrounding a tumor, dynamically interacts with the tumor, influencing growth, invasion, and metastasis. EVs facilitate this interaction by mediating intercellular exchanges through signaling pathways, enhancing communication between malignant and nonmalignant components of the TME, thus impacting antitumor responses [157]. Tumor-derived EVs (TEVs) affect both innate and adaptive immune responses by interacting with various immune cells such as lymphocytes, dendritic cells, macrophages, and myeloid-derived suppressor cells. These EVs carry tumor-specific antigens along with immunostimulatory and immunosuppressive molecules, leading to both antitumor and protumor effects. For instance, TEVs can mediate immunosuppression by transporting immunosuppressive cytokines like TGF-β and molecules such as FAS and PDL1, which can induce apoptosis in T cells and NK cells [158]. They also inhibit the maturation of dendritic cells and macrophages and suppress NK cell responses, facilitating tumor immune evasion [54,96]. Moreover, microRNAs within TEVs, such as mir-424, can suppress the CD28-CD80/86 co-stimulatory pathway, enhancing tumor immune suppression [135]. Conversely, tumor antigens presented by TEVs to T cells can activate antitumor responses, as demonstrated in studies with glioma-derived EVs in mice [159]. Additionally, TEVs can induce IFN-β production in dendritic cells through interactions with TLR3, influencing tumor progression and Treg cell dynamics [160]. Under stress conditions, tumor cells release altered EVs with a distinct molecular composition, carrying molecules like HMGB1, HSPs, ATP, and mitochondrial DNA, which create an inflammatory environment that aids in immune recognition of the tumor [96]. This aspect is crucial as cancer patients typically exhibit higher levels of EVs compared to healthy individuals, highlighting the potential of EVs as biomarkers for cancer diagnosis [59,161].

Focusing on CRC, the TME shows significant immunosuppressive potential, partly due to the inhibition of NK cell activation and chemotaxis by the TME architecture and signaling [135]. EVs also contribute to cancer drug resistance: for example, EVs from CD133+ cancer stem cells in CRC promote the tumorigenic capabilities of Cancer stem cells (CSCs) and inhibit the antitumor activity of drugs like oxaliplatin [162]. CRC-derived EVs release specific oncogenic miRNAs such as miR-21-5p and miR-200a, which mediate interactions between cancer cells and tumor-associated macrophages, driving tumor progression and metastasis [163]. Other miRNAs from CRC-associated EVs, like miR-25-3p and miR-130b-3p, stimulate cancer metastasis through the polarization of M2 macrophages, which are known for their protumor activities [163]. This encourages miRNAs from CRC EVs to be further investigated from the perspective of finding new treatments methods based on the inhibition of their secretion.

## 10. Therapeutic Potential

**Figure 2:**
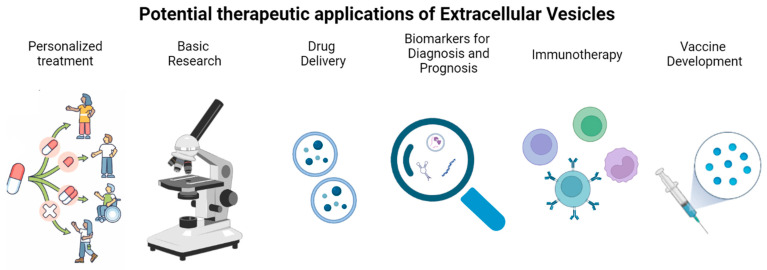
Potential therapeutic applications of EVs. This figure was created by the authors with BioRender.com, accessed on 16 May 2024.

The most significant insight from the existing research on EVs is their clinical relevance in diagnosing, monitoring, and treating various diseases, as well as their utility as biomarkers for identifying tumors and other pathological conditions, or as targets for therapeutic interventions. EVs serve as functional biomarkers in diverse medical fields, including organ transplants [164] (i.e., lung, heart, kidney, liver, and pancreas transplants), in diseases such as polymyositis, dermatomyositis [165], rheumatoid arthritis [166], and type 1 diabetes [167]. A notable study by Zhang et al. highlighted the potential of fecal EVs (fEVs) as biomarkers for CRC diagnosis and prognosis. Their study found that fEVs in CRC patients contained higher levels of CD147 and A33 proteins compared to healthy donors, effectively distinguishing between the two groups [168]. This specificity is particularly relevant for CRC due to fEVs’ unique relationship with the intestinal tract, unlike plasma EVs, which collect from various body districts and show negligible differences between healthy and cancer patients. Post-surgical observations have further confirmed the decrease in these markers, reinforcing their diagnostic and prognostic value [168]. Additionally, CAR–T cell-derived EVs show promise in cancer therapy. These EVs, capable of crossing biological barriers and targeting solid tumors without being affected by PD1-mediated immunosuppression, offer a more efficient alternative to CAR–T cells [96]. Stem cell-derived EVs also demonstrate significant immunotherapeutic potential, affecting various immune cells and processes in diseases like asthma and diabetes [169]. Furthermore, the potential of EVs extends to genetic engineering, where they can be tailored to carry therapeutic agents directly to tumor sites, enhancing drug delivery efficacy and targeting metastatic sites [54]. Data indicate that TEVs can serve as effective vehicles for drug delivery, leveraging their specific affinity to integrins in targeted tissues. This affinity enables these vesicles to efficiently distribute throughout the body, reaching metastatic tumor sites [170]. Lastly, EVs are being explored in vaccine development, using the outer membrane vesicles of Gram-negative bacteria as a platform. Cost-effective vaccines against pathogens like *Neisseria meningitidis* serogroup B have been made [171]. This simple and stable type of vaccine boasts several benefits: from its low production cost to the reduced risk of escape variants due to the ability to represent various antigenic molecules [96]. Figure 2 provides an overview of the therapeutic applications of EVs.

EV-based therapies face stringent regulatory pathways due to their novelty. Multiple clinical trials are currently investigating EVs as diagnostic, prognostic, or predictive biomarkers in CRC. The ExoColon trial evaluates circulating EV contents for their prognostic relevance in CRC, correlating with patient survival, cancer stage, and progression [172]. Trial NCT03432806 analyzes exosomes from pre-surgery peripheral blood in colon cancer patients to guide treatment decisions for colon and liver tumors [173]. NCT04394572 aims to identify new diagnostic protein markers for CRC by examining the number, size, and protein composition of blood exosomes [174]. Additionally, NCT03874559 investigates exosomal biomarkers in rectal cancer patients undergoing chemoradiation therapy, while NCT04227886 focuses on exosomal RNAs as predictive biomarkers for neoadjuvant chemoradiotherapy in rectal cancer [175,176]. One prospective feasibility study (NCT04852653) is assessing EVs obtained via liquid biopsy to monitor neoadjuvant treatment responses in CRC [177]. These trials encounter challenges such as standardizing EV isolation methods, ensuring adequate sensitivity and specificity, and achieving clinical-scale characterization. Additionally, EV-based therapies for CRC are primarily in early-phase trials without completed phase III studies, requiring more research to confirm their safety and efficacy. Concerns about off-target effects arise regarding EVs’ abilities to reach various tissues, potentially affecting non-target cells or organs. It is crucial to evaluate the immunogenicity risk of allogeneic or engineered EVs carefully.

## 11. Conclusions and Future Directions

Several important areas need further investigation to fully realize the therapeutic potential of EVs in cancer immunotherapy. Firstly, a deeper understanding of the molecular mechanisms governing the biogenesis, release, and uptake of EVs will be crucial for developing strategies to manipulate these processes for therapeutic purposes. This includes elucidating the role of specific miRNAs and proteins contained within EVs that contribute to immune modulation and tumor progression. Secondly, the development of more sophisticated methods for isolating and characterizing EVs is essential to ensure the purity and specificity of EV-based therapies. This will facilitate the identification of unique biomarkers for early cancer detection and monitoring treatment responses. Moreover, the potential of EVs as vehicles for targeted drug delivery presents a promising strategy for overcoming the limitations of current cancer therapies, such as non-specific toxicity and drug resistance. Engineering EVs to enhance their targeting efficiency and payload capacity could lead to more effective and less toxic treatments. Finally, clinical trials are imperative to validate the safety, efficacy, and therapeutic benefits of EV-based interventions in cancer patients. These studies should also explore the potential synergistic effects of combining EV-based therapies with existing treatment modalities, such as chemotherapy, radiation therapy, and checkpoint inhibitors. In conclusion, EVs represent a frontier in cancer immunotherapy, offering novel strategies for tumor targeting, immune modulation, and biomarker discovery. Continued research and innovation in this field holds the promise of transforming cancer treatment paradigms and improving patient outcomes.

## Figures and Tables

**Table 1:** Categorization of EVs based on primary characteristics.

Vesicles	Size (Diameter, nm)	Origin	Examples	Markers	References
Small-sized EVs	~50–150	Endosomes (exosomes); some from plasma membrane (ectosomes)	Exosomes, small ectosomes, ciliary ectosomes, microvesicles mediated by arrestin domain-containing protein 1	Tetraspanins, Alix, TSG101, CD63	[26,27,28,32,33,34]
Medium-sized EVs	~100–1000	Plasma membrane-derived ectosomes	Microvesicles, FDC-derived vesicles, T cell microvilli particles, elongated neutrophil-derived structures, secreted midbody remnants	Integrins, selectins, CD40	[26,27,28,35,36]
Large-sized EVs	~1000–5000	Plasma membrane-derived ectosomes, endoplasmic reticulum	Apoptotic bodies, large oncosomes, beaded apoptopodia, migrasomes, secretory autophagosomes	Phosphatidylserine, genomic DNA, receptors	[17,26,27,28,37,38,39]

**Table 2:** Comparison of methods for isolating and characterizing extracellular vesicles.

Isolation Methods	Purity	Principle	Advantages	Disadvantages	References
Ultracentrifugation	High	Differential centrifugation based on size and density	Large acquisition, relatively inexpensive	Time-consuming, may cause vesicle damage	[15,18,42,43]
Density-gradient centrifugation	High	Separation based on density differences	High purity, separates vesicle subpopulations	Labor-intensive, requires specialized equipment	
Immunoaffinity capture	High	Capture based on surface markers using specific antibodies	High specificity, allows for targeted isolation	High cost, limited by availability of specific antibodies	
Ultrafiltration	Moderate	Separation based on size differences	Relatively simple and rapid	Potential for vesicle damage, limited by pore size selection	
Precipitation	Low	Chemical or polymer-based precipitation of vesicles	High yield, relatively simple	Potential for co-precipitation of contaminants	

## Data Availability

No new data were created or analyzed in this study.

## Abbreviations

CAR, chimeric antigen receptor; EVs, extracellular vesicles; DCs, dendritic cells; NK, natural killer; NDTRs, neutrophil-derived trails; NDMVs, neutrophil-derived microvesicles; DAMPs, damage-associated molecular patterns; PRRs, pattern recognition receptors; ENDs, elongated neutrophil-derived structures; TME, tumor microenvironment; TEVs, tumor-derived EVs; CRC, colorectal cancer; fEVs, fecal EVs.
